# Acute Mesenteric Ischemia in a Chronically Anticoagulated Patient With Atrial Fibrillation: Anticoagulation Reversal, Management and Preventing Recurrence

**DOI:** 10.7759/cureus.21642

**Published:** 2022-01-26

**Authors:** Ahmed Ali Aziz, Donald Christmas

**Affiliations:** 1 Internal Medicine, Jersey Shore University Medical Center/St. Francis Medical Center, Trenton, USA

**Keywords:** anticoagulation failure, acute mesenteric ischemia, atrial fibrillation, direct oral anticoagulant therapy, arterial thromboembolism

## Abstract

Acute mesenteric ischemia (AMI) is caused by an interruption of the blood supply to the small intestine. Atrial fibrillation is a common cause of thromboembolic AMI. Patients taking direct oral anticoagulants (DOACs) for anticoagulation in atrial fibrillation are prone to anticoagulation failure and can present with thromboembolism. We present an interesting case of a 69-year-old female with a past medical history of chronic atrial fibrillation treated with a DOAC (apixaban) who was diagnosed with AMI despite being compliant with her anticoagulant. Her anticoagulation was promptly reversed and she was taken for urgent surgical intervention yielding a good outcome. Later, due to the failure of anticoagulation on apixaban her anticoagulant was changed to warfarin to prevent the recurrence of thromboembolism and follow-up showed she was doing well.

## Introduction

Acute mesenteric ischemia (AMI) is caused by an interruption of the blood supply to the small intestine which can be secondary to mesenteric arterial embolism (50%), mesenteric arterial thrombosis (15-25%), or mesenteric venous thrombosis (5-15%) [1]. It is a life-threatening emergency with a high mortality rate ranging between 50% and 70% [1]. Early diagnosis and intervention are necessary to reduce mortality [1]. Atrial fibrillation, heart failure, coronary heart disease, arterial hypertension, and peripheral vascular disease are common risk factors associated with AMI [1]. In particular, women with atrial fibrillation are at increased risk of thromboembolic events, including mesenteric ischemia [2]. Biphasic contrast-enhanced computed tomography angiogram is the diagnostic tool of choice to detect AMI. Patients should be managed in the intensive care unit and immediate surgical or endovascular intervention is warranted to reduce mortality. We present a unique case of a 69-year-old female with a past medical history of chronic atrial fibrillation taking apixaban for anticoagulation and end-stage renal disease (ESRD) on hemodialysis who developed AMI despite anticoagulation therapy. Her anticoagulation was promptly reversed and she was taken for urgent surgical intervention yielding a good outcome.

## Case presentation

**Figure 1:**
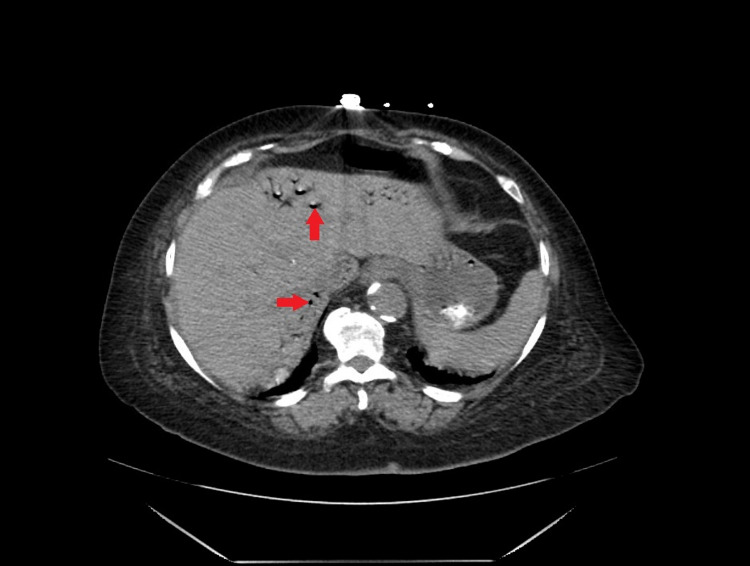
CT scan of abdomen and pelvis; red arrow points to air in portal venous vasculature.

**Figure 2:**
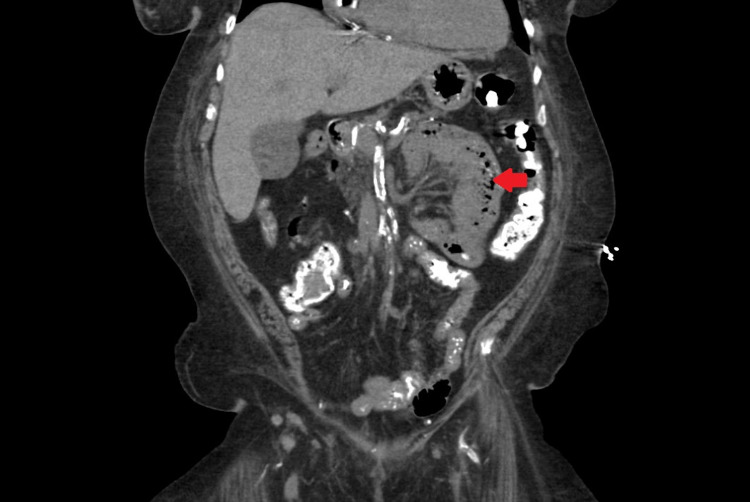
CT scan of abdomen and pelvis; red arrow points to small bowel pneumatosis.

A 69-year-old female with a past medical history of chronic atrial fibrillation taking apixaban for anticoagulation, hypertension, and ESRD on hemodialysis was admitted for a chief complaint of sudden onset of sharp abdominal pain and non-bloody vomiting. Her symptoms started a few hours prior to presenting to the hospital. On examination, her blood pressure was 128/62 mm Hg, pulse was 79 beats per minute, respiratory rate was 18 breaths per minute, she was afebrile and her O2 saturation was 100% on room air. Her abdomen was non-tender to palpation with normal bowel sounds. Initial lab work showed a normal white cell count, normal serum lactate levels, normal platelet count, and no increase in anion gap. Her hemoglobin was 11.1 g/dL, hematocrit 35.0%, international normalized ratio (INR) 1.3, prothrombin time (PT) 17.0 seconds and partial thromboplastin time (PTT) was 38.9 seconds. Computerized tomography (CT) scan of the abdomen and pelvis with IV contrast showed air in the portal and mesenteric vasculature as well as small bowel pneumatosis concerning for bowel ischemia (Figures 1, 2). The patient was admitted to the intensive care unit. Intravenous crystalloid fluid resuscitation and broad-spectrum antibiotics were initiated immediately. The patient’s last dose of apixaban was on the day of admission. Her anticoagulation was promptly reversed with prothrombin complex concentrate. She was taken for urgent exploratory laparotomy and 15 cm of necrotic distal ileum was resected. The edges of the transected intestine were viable and hence, an end-to-end and side-to-side anastomosis of the cut ends of the intestine was performed. Following surgery, the patient was started on total parenteral nutrition for six days for adequate bowel rest and was later advanced to a regular oral diet which she tolerated well. The patient was started on unfractionated heparin drip on postoperative day 3 and bridged to warfarin on postoperative day 13 for anticoagulation. Her anticoagulant was changed to warfarin to prevent the recurrence of thromboembolism. Follow-up two weeks and six months after discharge showed that she was doing well without any complaints.

## Discussion

Approximately 50% of cases of AMI result from mesenteric arterial cardioembolism [3, 4], and females with atrial fibrillation are at increased risk [2]. Hence, for patients who present with abdominal pain and have a history of atrial fibrillation, AMI should be on the list of differentials and should be ruled out as early as possible to reduce mortality.

AMI usually presents with a triad of acute onset severe abdominal pain, diarrhea or vomiting, and the presence of a known embolic source, like atrial fibrillation [5-7]. Patients describe pain that is out of proportion to physical exam findings [4, 8]. Common lab abnormalities include neutrophilic leukocytosis, hemoconcentration, lactic acidosis, and anion gap metabolic acidosis, however, these lab findings are not specific for AMI [9] and a normal physical exam or normal lab values should not deter the clinician away from considering AMI as a diagnosis.

Initial management requires intensive unit care, intravenous crystalloid fluid resuscitation and broad-spectrum antibiotics as patients with AMI are critically ill and can deteriorate at any moment secondary to bowel wall ischemia [1]. 

Anticoagulation failure on direct oral anticoagulants (DOACs) is not uncommon. Kajy, et al reported treatment failure with DOACs in their study [10] where the most common DOAC treatment failure was seen in patients with antiphospholipid syndrome (44.3%), atrial fibrillation (30.4%), and deep venous thrombosis (6.3%). Rivaroxaban had the highest failure rate (65.8%) followed by dabigatran (27.8%), apixaban (7.6%), and edoxaban (1.3%). Although our patient had AMI as a manifestation of DOAC treatment failure; for the above study, the most common manifestations of treatment failure were stroke/transient ischemic attack (20.3%), pulmonary embolism (19.0%), and deep venous thrombosis (19.0%) [10].

Patients with AMI on chronic anticoagulation require urgent anticoagulation reversal prior to surgical or endovascular intervention. Our patient was on apixaban which can be reversed using andexanet alfa. Andexanet alfa can also be used for rivaroxaban and edoxaban reversal while idarucizumab can be used for dabigatran reversal [11]. If the above agents are not available then prohemostatic agents like prothrombin complex concentrate (PCC) have also been used for DOAC reversal [11]. Our hospital pharmacy did not have andexanet alfa, hence we used PCC for DOAC reversal with a good outcome.

Immediate surgical or endovascular intervention is warranted if AMI is diagnosed to reduce mortality [1]. Surgery is preferred for patients with clinical signs of peritonitis, evidence of intestinal gangrene, central occlusion of the superior mesenteric artery, or when endovascular facilities are not available [1]. Endovascular techniques (angiographic catheter aspiration embolectomy or catheter lysis with recombinant tissue plasminogen activator) are gaining popularity in AMI treatment and are used in patients without clinical signs of peritonitis or intestinal gangrene [1]. Our patient had signs of intestinal gangrene on imaging and hence underwent surgical laparotomy and resection of the ischemic bowel.

For patients with anticoagulation failure on DOACs attempts should be made to identify the cause of failure. These include evaluating compliance issues, drug-drug or drug-food interactions [10]. If no underlying cause can be identified for DOAC failure then anticoagulation with a different DOAC or warfarin should be considered to prevent recurrence [10]. One of the reasons postulated for anticoagulation failure with DOACs is that DOACs specifically inhibit one of the coagulation factors (either factor II or X). This leads to the accumulation of upstream coagulation factors that may be enough to initiate a coagulation cascade in some individuals causing thrombus formation [10]. In contrast, the vitamin K antagonists act by inhibiting multiple coagulation factors (factors II, VII, IX and X), thereby effectively arresting the entire coagulation cascade [10]. Hence, our patient was transitioned to warfarin. Also, our patient had atrial fibrillation, ESRD and was on hemodialysis. Hu, et al [12] conducted a prevalence study of oral anticoagulation used by dialysis patients who had a history of atrial fibrillation and found that warfarin was the most commonly used oral anticoagulant in such patients [12]. Warfarin is preferred for use in ESRD because it is metabolized by the liver, not eliminated by kidneys, mostly bound to plasma proteins and is not filtered by dialysis hence efficacy is not altered by dialysis or ESRD [12]. In fact, warfarin was also the only oral anticoagulant recommended by the 2014 American Heart Association/American College of Cardiology/Heart Rhythm Society (AHA/ACC/HRS) guidelines for thromboembolic prophylaxis in ESRD patients with atrial fibrillation if their CHAD2DS2-VASc score was ≥ 2 [12]. Our patient had atrial fibrillation, ESRD and was on hemodialysis and as per above data warfarin was a suitable anticoagulant for her to prevent recurrence of thromboembolism. There are however no large scale trials that prove the benefits of a different DOAC or warfarin in preventing recurrence of thromboembolism in the event of DOAC failure.

## Conclusions

For patients who have a history of atrial fibrillation and present with abdominal pain, AMI should be on the list of differentials even if they are on anticoagulation. AMI should be ruled out earlier to decrease mortality. DOAC failure is not uncommon and anticoagulated patients can still develop thromboembolic phenomena. To prevent recurrence of thromboembolism in patients with DOAC failure, anticoagulation should be pursued with a different DOAC with an alternate mechanism of action or warfarin should be considered.
